# Machine learning-based prediction of bloodstream infection in rectal carbapenem-resistant *Enterobacterales* carriers: a multicenter retrospective cohort study

**DOI:** 10.3389/fcimb.2026.1791859

**Published:** 2026-08-06

**Authors:** Kibum Jeon, Wonkeun Song, Dong Hoon Shin, Han-Sung Kim, Hyun Soo Kim, Jacob Lee, Seok Hoon Jeong, Seri Jeong

**Affiliations:** 1 Department of Laboratory Medicine, Hallym University Hangang Sacred Heart Hospital, Hallym University College of Medicine, Seoul, Republic of Korea; 2 Department of Laboratory Medicine, Hallym University Kangnam Sacred Heart Hospital, Hallym University College of Medicine, Seoul, Republic of Korea; 3 Department of Laboratory Medicine, Hallym University Chuncheon Sacred Heart Hospital, Hallym University College of Medicine, Chuncheon, Republic of Korea; 4 Department of Laboratory Medicine, Hallym University Sacred Heart Hospital, Hallym University College of Medicine, Anyang, Republic of Korea; 5 Department of Laboratory Medicine, Hallym University Dongtan Sacred Heart Hospital, Hallym University College of Medicine, Hwaseong, Republic of Korea; 6 Department of Internal Medicine, Hallym University Kangnam Sacred Heart Hospital, Hallym University College of Medicine, Seoul, Republic of Korea; 7 Department of Laboratory Medicine, Gangnam Severance Hospital, Yonsei University College of Medicine, Seoul, Republic of Korea

**Keywords:** bloodstream infection, carbapenem-resistant *Enterobacterales*, explainable artificial intelligence, machine learning, risk prediction, XGBoost ML models

## Abstract

**Background:**

Carbapenem-resistant *Enterobacterales* (CRE) carriers are at risk of developing subsequent CRE bloodstream infection (BSI). This study aimed to identify features contributing to BSI development in rectal CRE carriers and interpret their interactions using machine learning (ML) and explainable artificial intelligence (XAI), while implementing individualized BSI risk prediction.

**Methods:**

We included patients with first-time positive rectal CRE surveillance cultures during hospitalization at three hospitals in Korea from January 2014 to December 2023. A total of 173 features—laboratory data including the carbapenem minimum inhibitory concentrations (MIC) of rectal CRE isolates and complete blood cell count, demographics, vital signs, comorbidities, medications, transfusions, and procedures —were collected for the 30-day and 14-day follow-up periods, of which 151 remained after removing redundant features. The entire dataset was randomly split into training (80%) and test (20%) sets, and XGBoost ML models were developed. Model performance was evaluated using the area under the receiver operator characteristic curve (AUROC) with 1, 000 bootstrap iterations. SHAP (SHapley Additive exPlanations) was employed as the XAI method.

**Results:**

Among 639 included CRE carriers, 72 (11.3%) and 46 (7.2%) developed BSI within 30 and 14 days, respectively. The 30-day and 14-day models demonstrated excellent performance with AUROCs of 0.917 (95% CI: 0.889-0.943) and 0.854 (95% CI: 0.821-0.885), respectively. In the 30-day model, the most contributory feature was minimum inhibitory concentration (MIC) of imipenem or meropenem against rectal CRE, with BSI risk increasing when MIC ≥8 μg/mL. In the 14-day model, increased neutrophil count (≥7.50×10³/μL) was the most contributory feature. Several features showed nonlinear relationships with BSI risk and contrasting contributions depending on feature interactions. Individual 30-day and 14-day prediction models required at least 12 top-contributing features, achieving AUROCs of 0.873 (95% CI: 0.728-0.969) and 0.864 (95% CI: 0.727-0.959), respectively.

**Conclusions:**

The influence of contributing features varied by time periods and interactions. This study comprehensively interpreted complex factors using ML and XAI, achieving excellent predictive performance. Individualized BSI risk stratification can facilitate determining appropriate timing for preemptive therapy and inform antimicrobial stewardship decisions.

## 1 Introduction

Carbapenem-resistant *Enterobacterales* (CRE) have emerged as a major global public health threat, posing a significant challenge to infection control within healthcare settings. In Korea, high-risk patients upon admission or those who have had contact with CRE-infected patients undergo surveillance screening. CRE colonization is a risk factor for subsequent infection, with a meta-analysis reporting a cumulative infection rate of 16.5% in CRE carriers (Tischendorf et al., 2016). Among CRE infections, bloodstream infection (BSI) is associated with significantly increased mortality (Capone et al., 2013**;** Viale et al., 2013). While timely antimicrobial treatment can reduce CRE-BSI mortality (Falcone et al., 2020), evidence supporting decolonization therapy to prevent BSI in asymptomatic carriers remains limited (Mascolo et al., 2023). Therefore, accurately predicting subsequent BSI risk in CRE carriers identified through rectal surveillance cultures could enable targeted interventions, improve infection control, and prevent unnecessary antibiotic use.

Previous studies on CRE-BSI risk prediction have relied predominantly on traditional linear models (Chu et al., 2022**;** Giannella et al., 2014**;** Liu et al., 2023). However, the development of BSI involves non-linear interactions among host immunity, comorbidities, microbiological characteristics, and hospital-related factors. Traditional linear models cannot adequately capture these intricate relationships, resulting in limited predictive accuracy (Lundberg and Lee, 2017). Furthermore, inconsistent findings across studies have hindered the widespread clinical application of existing predictive models.

Machine learning (ML) has been increasingly adopted in clinical research and has demonstrated superior performance in handling high-dimensional clinical data and modeling non-linear relationships (De Mutsert et al., 2011**;** Saranya and Subhashini, 2023). Despite their predictive power, ML models are often criticized as “black boxes.” To address this, Explainable Artificial Intelligence (XAI) techniques, such as SHAP (SHapley Additive exPlanations), have been developed to provide transparency by quantifying the contribution of each feature to individual prediction (Jahmunah et al., 2022**;** Lundberg et al., 2020**;** Meena and Hasija, 2022**;** Miró-Nicolau et al., 2022).

This study had two primary objectives: first, to develop and validate ML models —using XGBoost with SHAP-based XAI—that identify features contributing to subsequent CRE-BSI in rectal CRE carriers and interpret their interactions across the 30-day and 14-day follow-up periods; second, to implement an individualized CRE-BSI risk prediction model based on routinely available demographic and laboratory data to support clinical decision-making. Unlike previous studies limited to a single CRE species or a specific patient population, this study applies ML with XAI to predict CRE-BSI across all CRE species, drawing on routine laboratory test data collected from a 10-year multicenter cohort. By making the basis of each prediction interpretable, this approach is intended to provide individualized guidance for preemptive therapy and antimicrobial stewardship in clinical practice.

## 2 Materials and methods

### 2.1 Study design and population

This retrospective cohort study was conducted across three hospitals affiliated with Hallym University Medical Center from January 1, 2014, to December 31, 2023. The study population comprised adult patients (≥18 years) whose first-ever rectal CRE colonization was identified during hospitalization. Exclusion criteria were: (1) had CRE-BSI prior to rectal culture collection, (2) died or were discharged within the 30-day follow-up period, (3) had concurrent CRE infection at another anatomical site (e.g., pneumonia or urinary tract infection), (4) had incomplete data with missing values in collected features, (5) had a length of hospital stay ≤48 hours, or (6) had a positive rectal CRE surveillance culture within 24 hours of hospital admission.

### 2.2 Data collection and definitions

Data were extracted from the Clinical Data Warehouse (CDW). We collected 173 features within seven days prior to or on the date of the rectal culture collection, including demographics, vital signs, comorbidities, laboratory tests (including microbiology, complete blood count, coagulation studies, and chemistry panels), medications, transfusions, and procedures; all 173 features are listed in **Supplementary Table 1**. Of these features, the minimum inhibitory concentration (MIC) is the lowest antimicrobial concentration that inhibits visible growth of an isolate; the imipenem or meropenem MIC against the rectal CRE isolate (μg/mL) was determined using the Vitek 2 (bioMérieux, USA) or MicroScan Walkaway-96 system (Siemens, USA). Additional CRE colonization denoted a concurrent CRE-positive culture in other sample; hypertension was defined as a documented physician diagnosis; and age was recorded in years.The primary outcome was the first episode of CRE-BSI occurring within 14 or 30 days after the first positive rectal CRE culture. All patients included were followed up for the 14 and 30-day periods without being divided into separate follow-up groups. Accordingly, two parallel models were developed on the same cohort using the same baseline features, differing only in the outcome window (CRE-BSI within 14 versus 30 days). CRE-BSI was defined as the isolation of a CRE from one or more blood cultures in a patient with clinical signs of infection; the date of BSI was defined as the collection date of the first positive blood culture. CRE-BSI risk was predicted from the time of the first positive rectal CRE surveillance culture, for two follow-up periods (14 and 30 days). CRE was defined according to the Clinical and Laboratory Standards Institute (CLSI) guidelines M100S as follows: (1) resistance to one or more carbapenems (imipenem, meropenem, or doripenem MIC ≥4 mg/L, or ertapenem MIC ≥2 mg/L); (2) presence of a carbapenemase-producing organism; or (3) for intrinsically imipenem-resistant organisms (*Morganella morganii*, *Proteus* spp., and *Providencia* spp.), resistance to one or more meropenem, ertapenem, or doripenem (Clinical and Laboratory Standards Institute, 2024).

The identification of the CRE isolates was performed using the Vitek 2 system (bioMérieux, USA), MicroScan Walkaway-96 system (Siemens, USA), or matrix-assisted laser desorption ionization-time-of-flight mass spectrometry (Vitek MS, bioMérieux). Antimicrobial susceptibility test and MICs for the CRE isolates were determined using the Vitek 2 system or MicroScan Walkaway-96 system. Carbapenemase presence was screened via NG-Test CARBA 5 immunochromatographic assay (NG Biotech, France) and confirmed by multiplex real-time PCR (Panagene, Korea).

### 2.3 Model development and validation, and statistical analysis

The dataset was randomly divided into training (80%) and test (20%) sets. We compared three algorithms: logistic regression, deep neural network (Rajkomar et al., 2019), and XGBoost (Chen and Guestrin, 2016). Optimal hyperparameters were determined through 5-fold cross-validation using GridSearchCV (Belete and Huchaiah, 2022).

Model performance was evaluated via the area under the receiver operating characteristic curve (AUROC) and area under the precision–recall curve (AUPRC). To assess the statistical significance of the performance differences between the models and calculate 95% confidence intervals, we performed 1, 000 bootstrap iterations on the test set. Model performance based on AUROC was interpreted as follows: 0.7 to 0.8, acceptable; 0.8 to 0.9, excellent; and >0.9, outstanding (Mandrekar, 2010). Continuous variables are summarized as medians (range) and categorical variables as numbers (percentages). All analyses were performed using Python 3.11.5 with the scikit-learn (version 1.2.2) and SHAP (version 0.44.1) libraries, and significance was set at p-value < 0.05.

### 2.4 Model explainability and feature selection

SHAP with TreeExplainer (version 0.44.1) (Lundberg et al., 2020) were employed as XAI methodologies and generated both global and local explanations of model predictions.

To construct an optimal model and reduce feature redundancy, we hierarchically clustered the 173 features based on SHAP values, using a distance metric ranging from 0 (perfect redundancy) to 1 (complete independence) (Cooper et al., 2021). Within perfectly redundant clusters, we retained only the feature with the highest SHAP value and removed the others. The remaining features were used for the subsequent analysis (**Supplementary Figure 1**).

To develop a simplified individualized CRE-BSI risk model, we employed recursive feature elimination (RFE). From the features retained after redundancy removal, we selected 87 demographic and laboratory test features for this model. In each iteration, we ranked the features by their mean absolute SHAP values and removed the five least important features. We then trained a new model on the remaining feature set and repeated this cycle until the target number of features was achieved. To evaluate predictive performance, we bootstrapped the test set 1, 000 times and reported the mean AUROC with 95% confidence intervals. All feature-selection steps (hierarchical clustering and recursive feature elimination) were performed within the training set to prevent information leakage.

## 3 Results

### 3.1 Study population and CRE-BSI incidence

**Figure 1:**
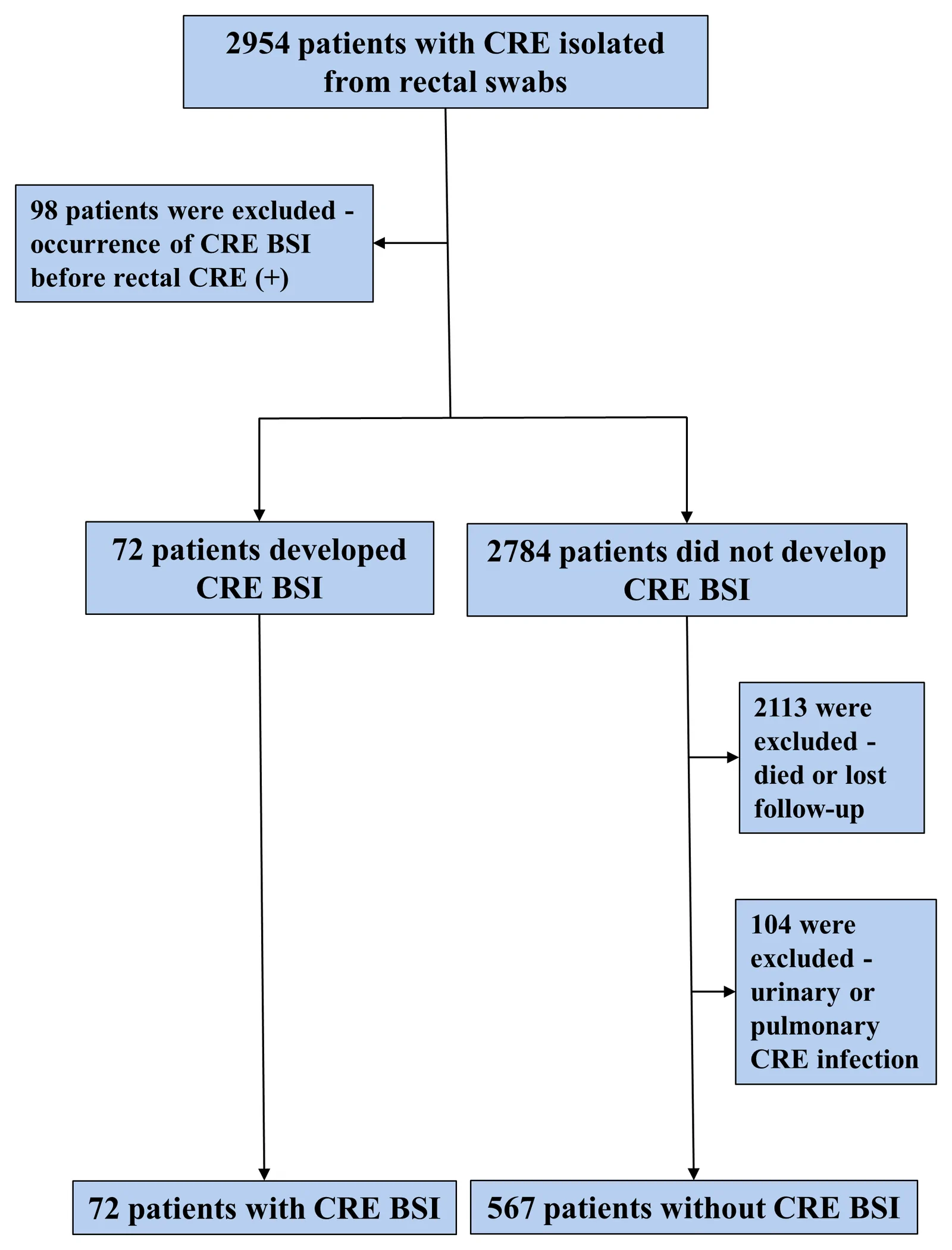
Flow chart for subject selection. CRE BSI, Carbapenem-resistant *Enterobacterales* bloodstream infection.

From 2, 954 initial positive screenings during the 10-year study period, 639 patients met the inclusion criteria (**Figure 1**). Among these, 11.3% (72/639) developed CRE-BSI within 30 days, and 7.2% (46/639) within 14 days. The median time to BSI was 11 days (range: 1–29 days). The 30-day mortality rate among patients with CRE-BSI was 63.9%.

### 3.2 Microbiological characteristics

CRE species from rectal surveillance cultures included: *K. pneumoniae* in 471 patients (73.7%), *E. coli* in 69 (10.8%), *Enterobacter* species in 59 (9.2%), other *Klebsiella* species in 27 (4.2%), *S. marcescens* in 12 (1.9%), *Citrobacter* species in 9 (1.4%), and *Kluyvera* and *Raoultella* species in one patient each.

Among 72 patients with CRE-BSI, blood culture isolates included: *K. pneumoniae* (N = 52, 70.3%), *Enterobacter* spp. and *S. marcescens* (N = 6 each, 8.3%), *E. coli* (N = 4, 5.6%), and *K. oxytoca* (N = 2, 2.8%). Concordance between rectal surveillance and blood culture isolates was observed in 61 patients (84.7%).

Antimicrobial resistance rates showed high concordance between rectal and blood isolates. Resistance rates for imipenem (71.4%), ertapenem (100%), and amikacin (9.7%) were identical. Similar patterns were observed for meropenem (rectal: 95.0%, blood: 93.3%), tigecycline (50.7% vs. 37.7%), levofloxacin (89.8% vs. 91.5%), aztreonam (98.6% vs. 97.2%), and piperacillin-tazobactam (100% vs. 98.6%).

Carbapenemase-producing *Enterobacterales* (CPE) were identified in 279 patients (43.7%). The distribution of carbapenemase types was as follows: *KPC* in 145 patients (52.0%), *NDM* in 84 (30.1%), and *OXA-48*-*like* in 52 (18.6%).

### 3.3 ML model selection and performance

**Figure 2:**
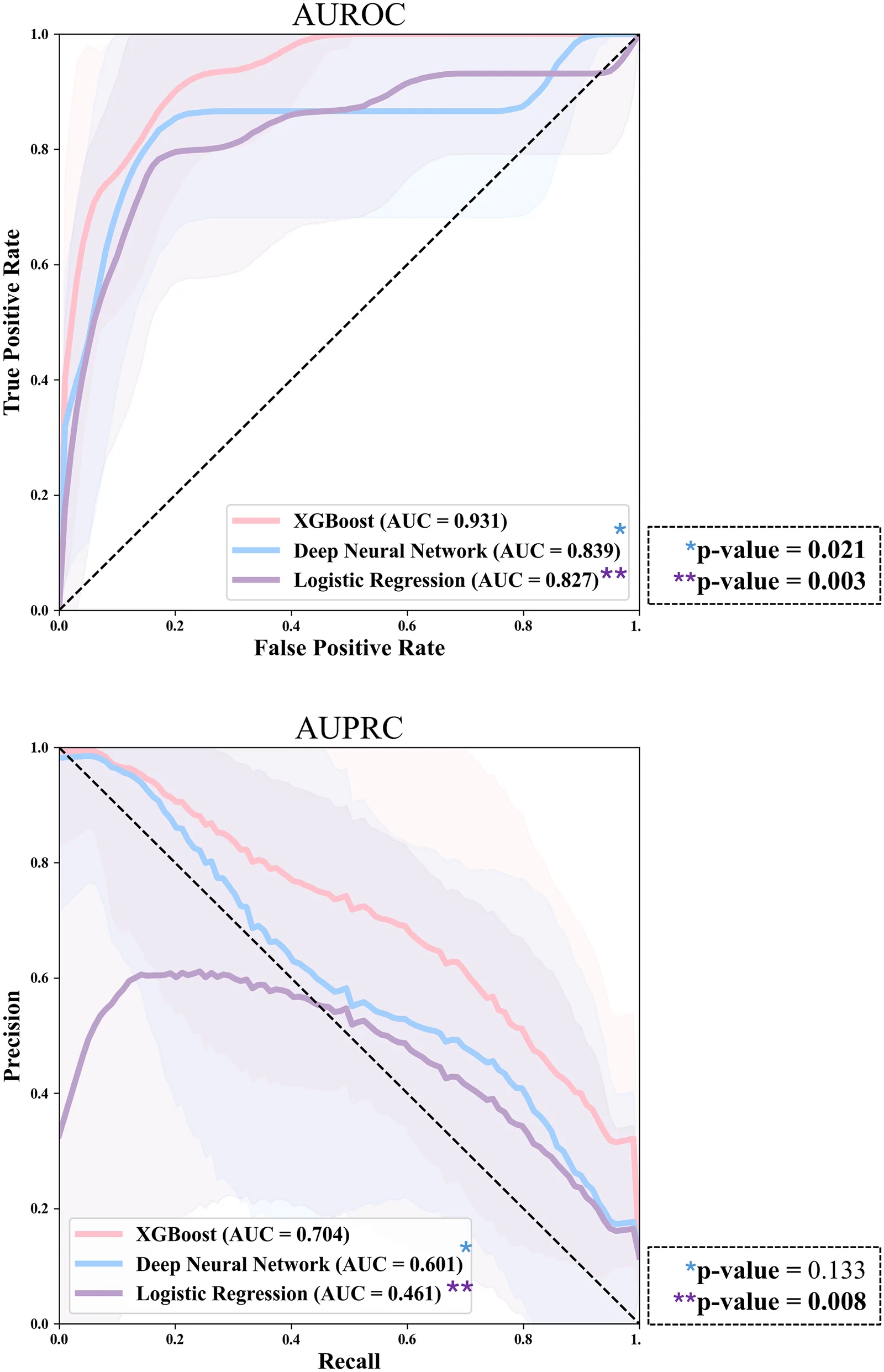
Performance comparison of three machine learning models. The solid lines indicate the mean area under the receiver operating characteristic curve (AUROC) and area under the precision-recall curve (AUPRC) values derived from 1, 000 bootstrap iterations. The shaded regions indicate 95% confidence intervals.

The XGBoost model achieved an AUROC of 0.931, indicating outstanding performance. It significantly outperformed logistic regression (AUROC: 0.827; p-value = 0.003) and deep neural network (AUROC: 0.899; p-value = 0.021), and was selected as the final model (**Figure 2**).

After supervised clustering to address feature redundancy, the final model utilized 151 features. The 30-day CRE-BSI prediction model achieved an AUROC of 0.917 (95% CI: 0.889-0.943), while the 14-day model achieved an AUROC of 0.854 (95% CI: 0.821-0.885).

### 3.4 Feature importance analysis by prediction period

**Figure 3:**
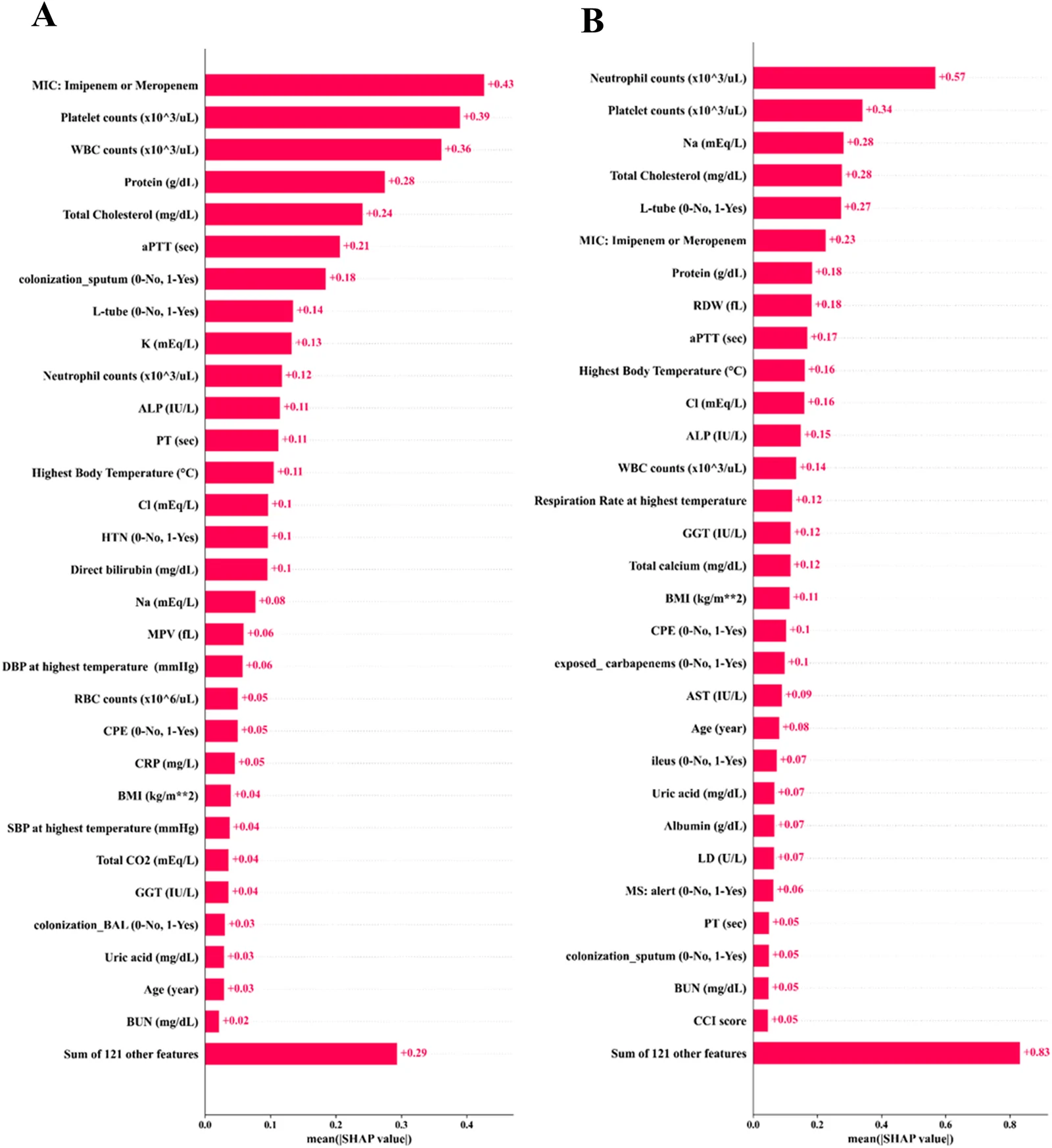
Global feature importance plot for the CRE-BSI prediction model. **(A)** 30-day prediction model. **(B)** 14-day prediction model. The features are displayed in descending order on the basis of their mean absolute SHAP values, indicating each feature’s average contribution to model predictions.

For the 30-day CRE-BSI prediction model, imipenem or meropenem MIC values from rectal CRE isolates emerged as the most significant contributory feature, followed by platelet count and white blood cell (WBC) count. Additional important features included serum protein, total cholesterol, activated partial thromboplastin time (aPTT), and additional CRE colonization in sputum (**Figure 3A**). Among the top 30 features, 23 (76.7%) were laboratory tests, while non-laboratory features included L-tube insertion, highest body temperature, hypertension, blood pressure at highest temperature, body mass index (BMI), and age.

In the 14-day model, neutrophil count emerged as the most significant contributory feature, followed by platelet count, sodium levels, total cholesterol, imipenem or meropenem MIC, and L-tube insertion (**Figure 3B**).

Forty-five features showed zero SHAP values in both models, indicating no predictive contribution (**Supplementary Table 2**). These included chemotherapy—previously reported as a common risk factor (Chu et al., 2022**;** Giannella et al., 2014)—invasive abdominal procedures, and admission during the COVID-19 pandemic.

### 3.5 Feature impacts vary by prediction period

**Figure 4:**
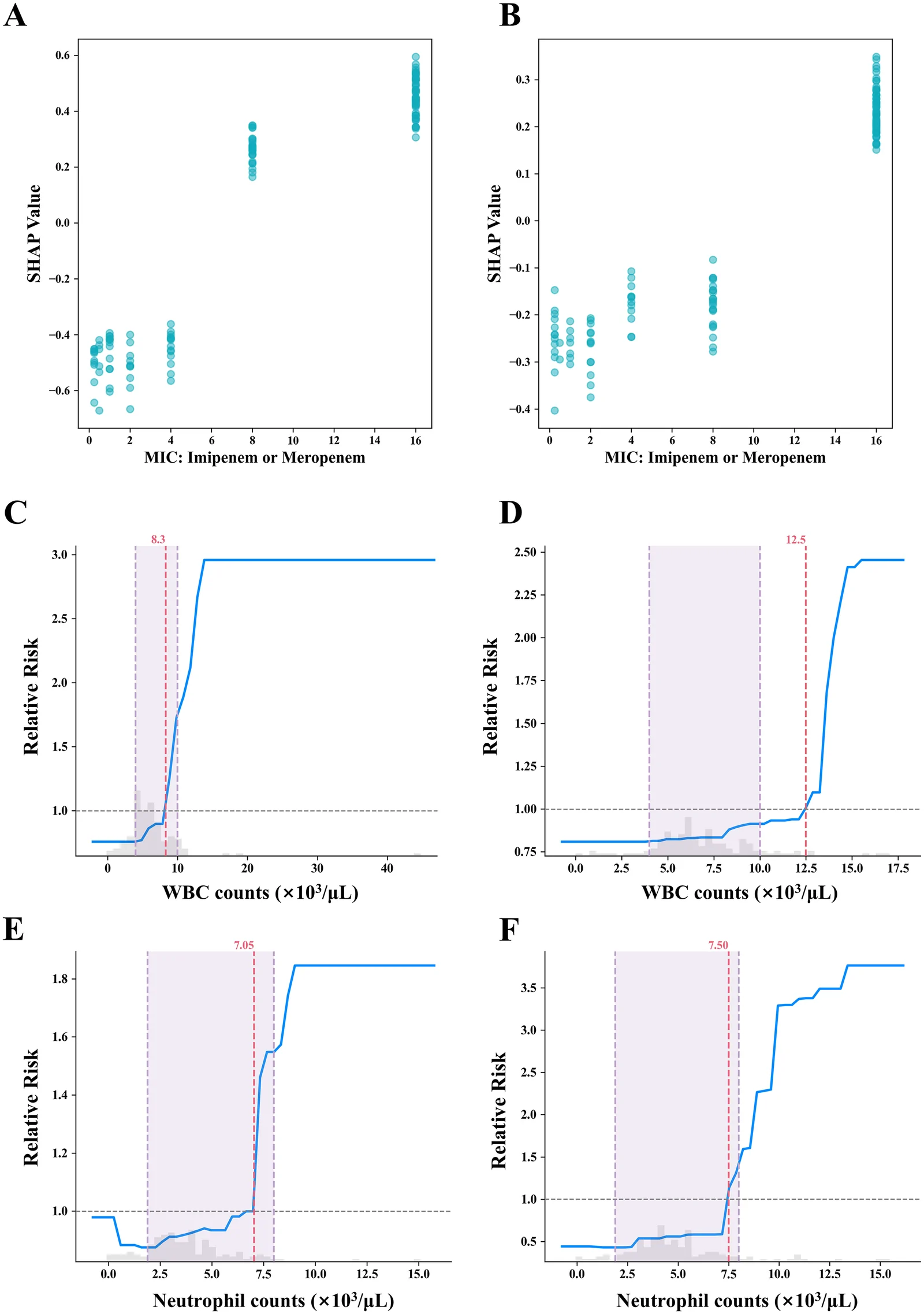
Comparison of feature impacts according to the prediction period. **(A, B)** SHAP value plot. Each point represents an individual patient; the x-axis indicates the feature value (MIC), and the y-axis indicates the corresponding SHAP value. **(A)** Imipenem or meropenem MIC of rectal CRE isolates in the 30-day prediction model. **(B)** Imipenem or meropenem MIC in the 14-day prediction model. **(C-F)** SHAP dependence plots for CRE bloodstream infection (BSI) risk. X-axis represents the feature value, and the y-axis represents the relative risk of BSI. Purple shading indicates the reference range; the red dashed line indicates the threshold (cut-off value) where the relative risk exceeds 1, indicating an increased risk of BSI; the gray histogram represents the distribution of feature values. **(C)** WBC count in the 30-day prediction model. **(D)** WBC count in the 14-day prediction model. **(E)** Neutrophil count in the 30-day prediction model. **(F)** Neutrophil count in the 14-day prediction model.

The contributions of individual features to CRE-BSI prediction differed between the 30-day and 14-day models. SHAP value plots for imipenem or meropenem MICs of rectal CRE isolates (**Figures 4A, B**) showed that risk increased from MIC ≥8 μg/mL in the 30-day model, whereas the 14-day model required a higher threshold of ≥16 μg/mL.

For WBC counts (**Figures 4C, D**), the 30-day prediction showed increased risk when exceeding 8.3×10³/μL, while the 14-day prediction required exceeding 12.5×10³/μL.

Neutrophil count thresholds (**Figures 4E, F**) showed minimal differences: 7.05×10³/μL (30-day) versus 7.50×10³/μL (14-day). However, maximum risk increases differed substantially—83% in the 30-day model versus 290% in the 14-day model, demonstrating more than threefold elevation in the shorter prediction period.

### 3.6 Interaction effects of features

**Figure 5:**
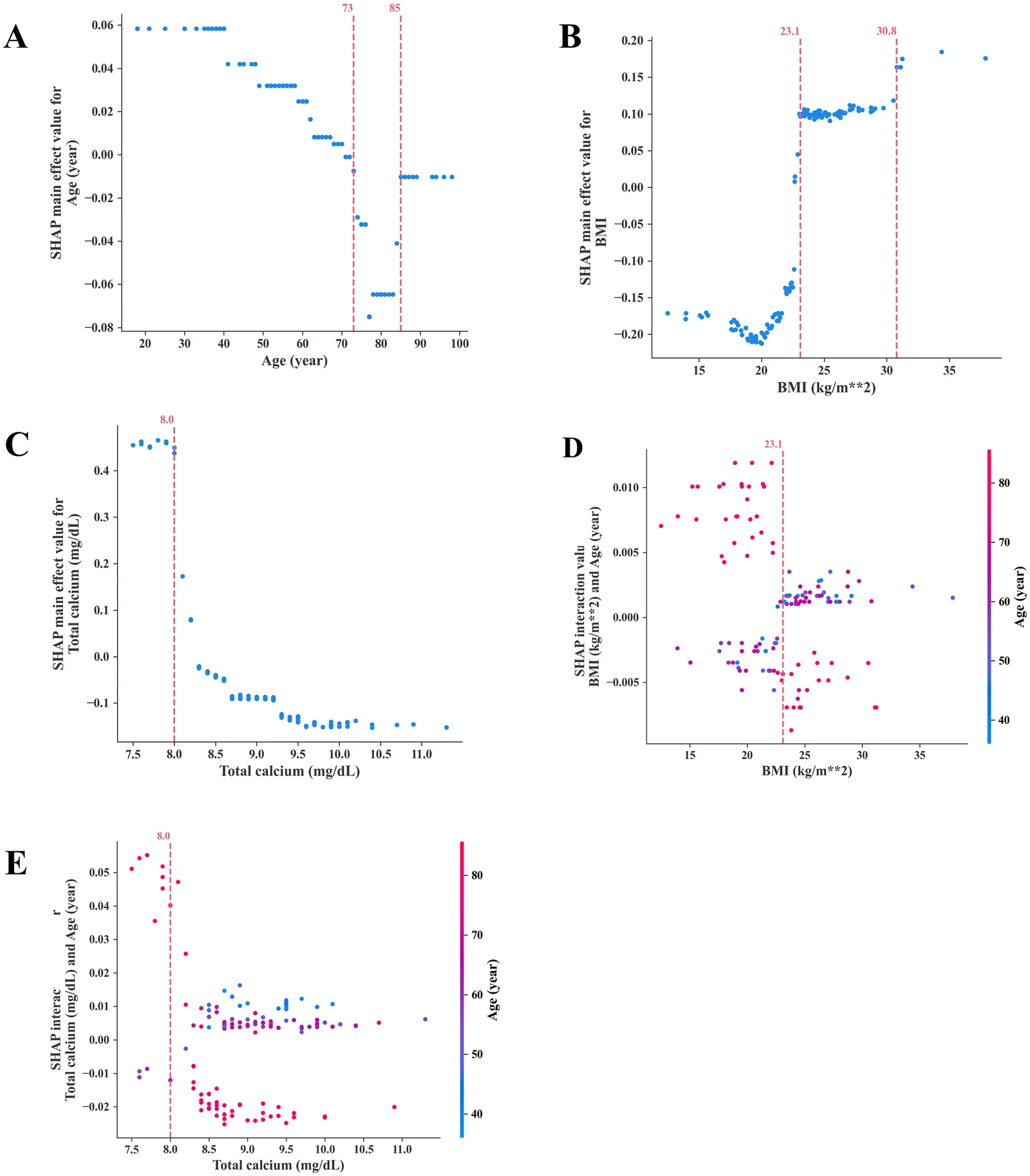
Interaction effects of features with age. **(A–C)** SHAP main effect plots. The SHAP main effect represents the isolated contribution of a specific feature to the model prediction, calculated by subtracting interaction values from the total SHAP value. Each point represents an individual patient; the x-axis indicates the feature value, and the y-axis indicates the SHAP main effect value. **(A)** Age; **(B)** Body mass index (BMI); **(C)** Total calcium. (**D–E)** SHAP interaction effect plots. These plots illustrate how the impact of specific features on the risk of CRE bloodstream infection varies across different ages. Each point represents a single patient, with the color gradient indicating age (redder points represent older patients). **(D)** Interaction between BMI and age; **(E)** Interaction between total calcium and age.

Age demonstrated a U-shaped relationship with BSI risk: risk decreased until approximately 73 years and increased beyond 85 years (**Figure 5A**). BSI risk increased with BMI ≥23.1 kg/m² and further increased with BMI ≥30.8 kg/m² (**Figure 5B**). Serum calcium showed sharp risk increase when levels fell below 8.0 mg/dL (**Figure 5C**).

The interaction between age and BMI (**Figure 5D**) revealed that when BMI was ≥23.1 kg/m², BSI risk was greater in middle-aged patients than in elderly patients. Conversely, the calcium-age interaction (**Figure 5E**) showed the opposite pattern: when serum calcium was <8.0 mg/dL, BSI risk was greater in elderly patients.

### 3.7 Simplified individual risk prediction models

**Figure 6:**
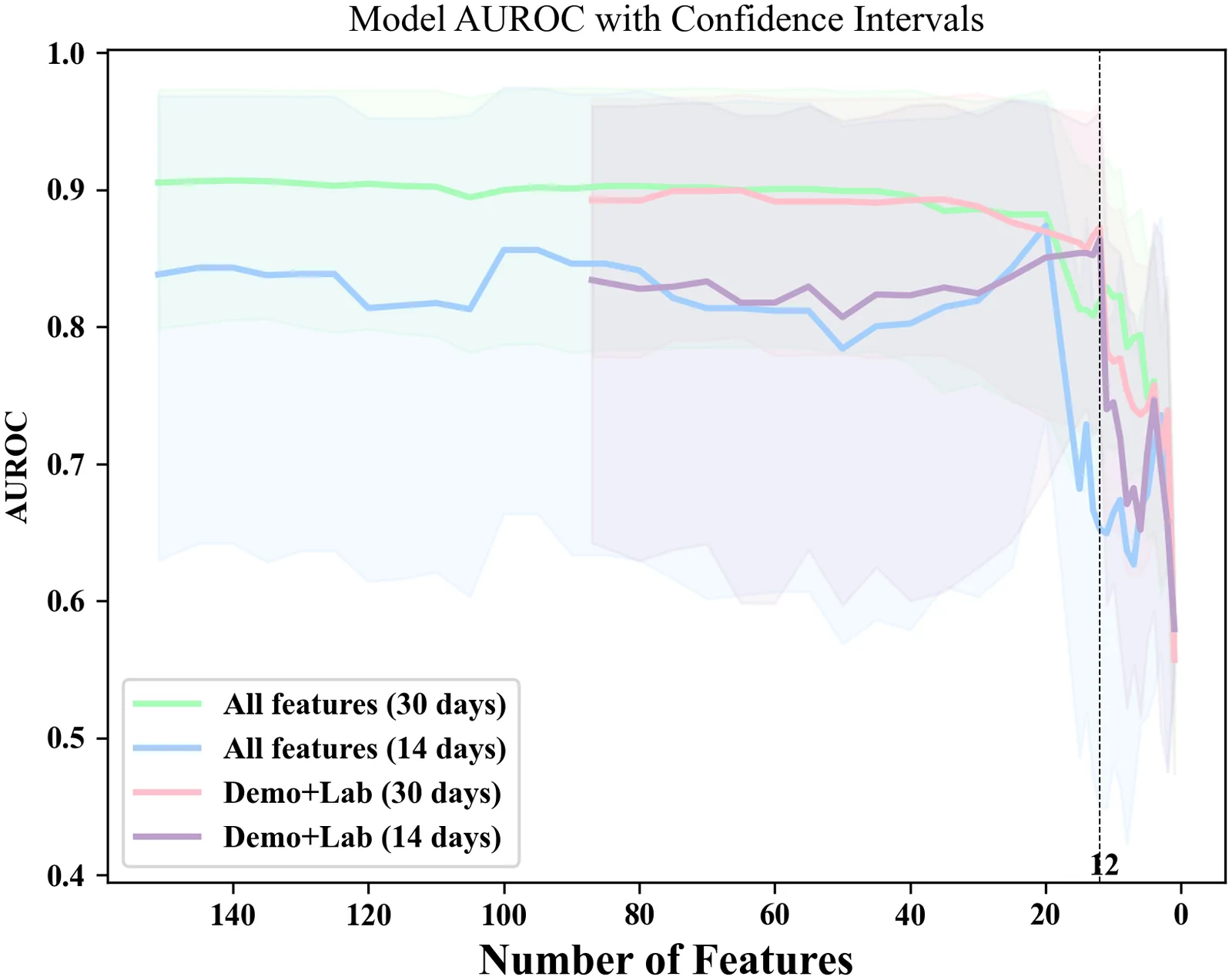
Model performance during recursive feature elimination across different feature sets. Lines represent the mean area under the receiver operating characteristic curve (AUROC) values derived from 1, 000 bootstrap iterations; shaded areas represent the 95% confidence intervals. “All features” refers to the model trained using the 151 features; “Demo+Lab” refers to the model trained using 87 demographic and laboratory test features.

Using recursive feature elimination, we identified the minimum feature set required to maintain an AUROC above 0.850, corresponding to excellent discrimination performance (Mandrekar, 2010). Both the 30-day and 14-day models achieved this threshold with 12 features, yielding AUROC values of 0.873 (95% CI, 0.728–0.969) and 0.864 (95% CI, 0.727–0.959), respectively (**Figure 6**).

**Figure 7:**
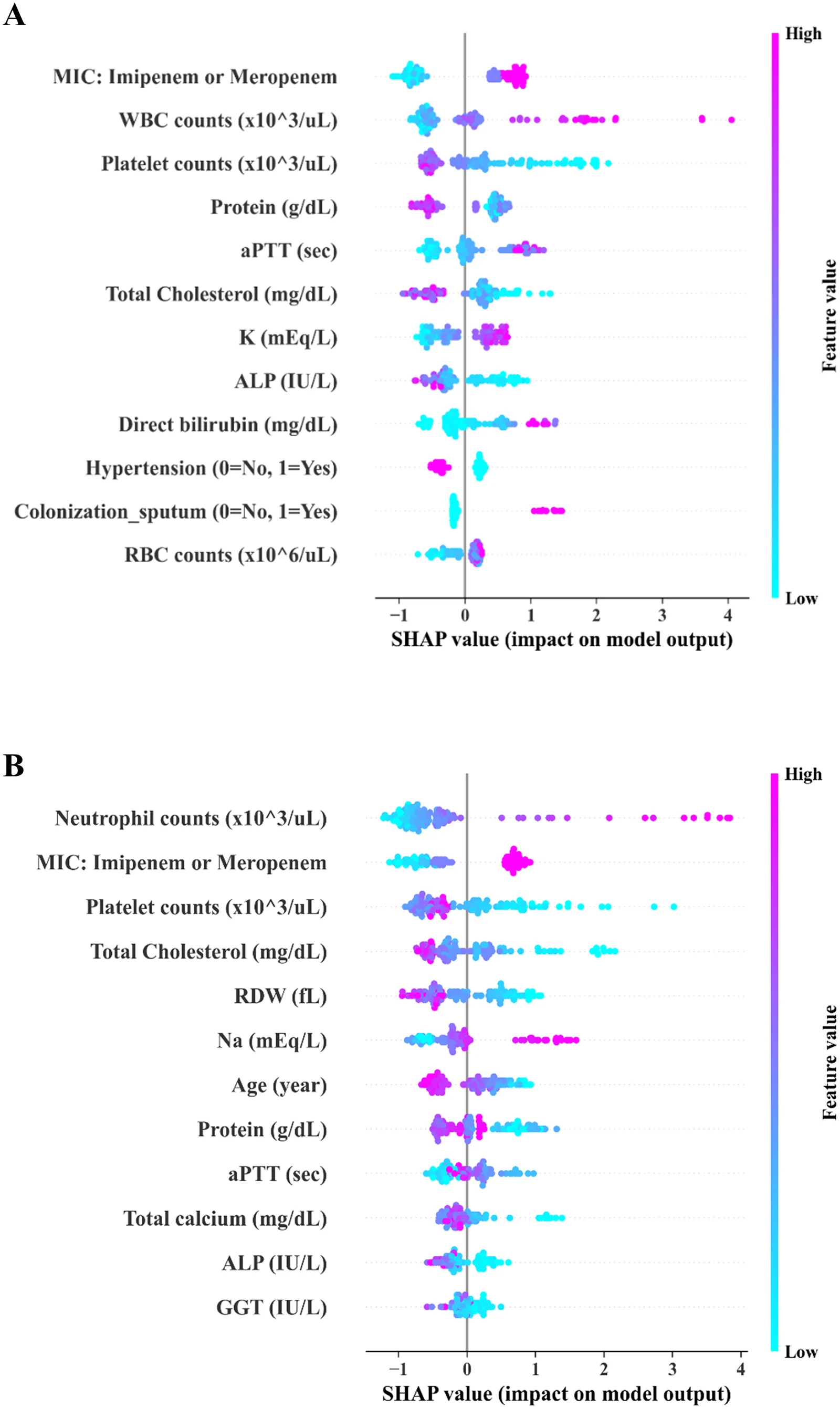
SHAP summary plot for the simplified CRE-BSI prediction model. **(A)** 30-day prediction model. **(B)** 14-day prediction model. The plot displays the top 12 features ranked in descending order of their importance. Each point represents an individual patient; the color represents the feature value (red/magenta indicates high values, blue/cyan indicates low values). The horizontal spread of the points (beeswarm distribution) reflects the density of samples at a specific SHAP value. The x-axis indicates the SHAP value: positive values (to the right of the zero line) denote a contribution toward an increased risk of BSI, whereas negative values (to the left) denote a contribution toward a decreased risk.

For the 30-day prediction model, selected features in order of contribution were imipenem or meropenem MIC, WBC count, platelet count, protein, aPTT, total cholesterol, potassium, ALP, direct bilirubin, hypertension, additional CRE colonization in sputum, and RBC count (**Figure 7A**). The 14-day model included the neutrophil count, imipenem or meropenem MIC, platelet count, total cholesterol, RDW, sodium, age, protein, aPTT, calcium, ALP, and GGT (**Figure 7B**).

**Figure 8:**
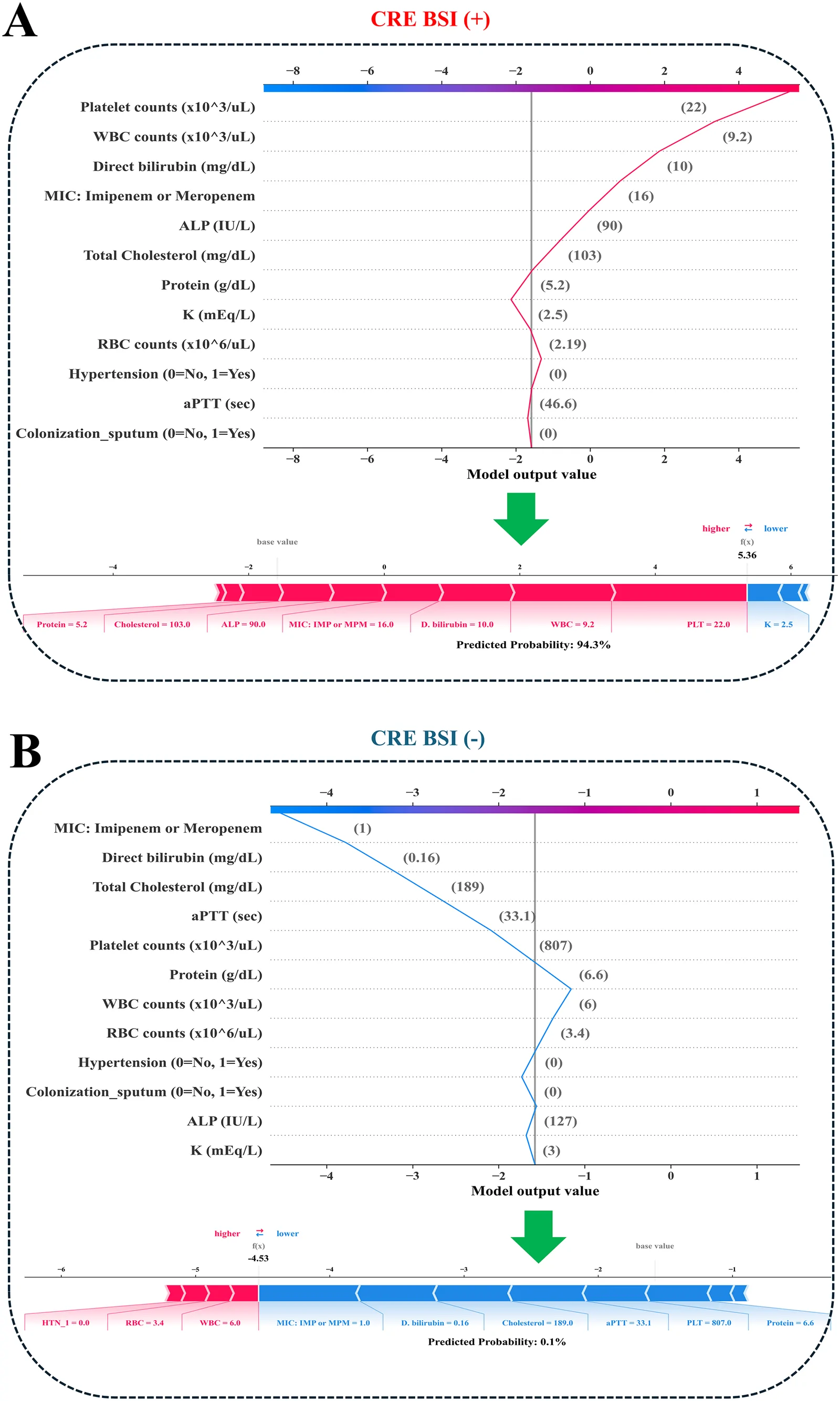
Individualized SHAP explanations of simplified CRE-BSI prediction model. **(A)** Representative case of a patient who developed CRE-BSI. The top panel presents a SHAP decision plot showing cumulative log-odds contributions from features ordered by their impact, ranging from CRE colonization in sputum to the most influential feature (platelet count). Actual feature values are provided in parentheses. Starting from the baseline (expected value), each feature progressively shifts the prediction; platelet count (22.0×10³/μL) provides the strongest positive contribution toward a rightward shift. The bottom panel displays a SHAP force plot, where red bars indicate features increasing the risk of BSI and blue bars indicate features decreasing the risk. The cumulative effect yielded a log-odds of 5.36, corresponding to a 94.3% BSI probability. **(B)** Representative case of a patient who did not develop CRE-BSI. The top panel shows a SHAP decision plot, where a low imipenem or meropenem MIC (1 μg/mL) provided the strongest negative contribution. The bottom panel displays the corresponding SHAP force plot; the cumulative effect resulted in a log-odds of -4.53, corresponding to a 0.1% BSI probability.

**Figure 8A** shows the prediction for a patient who subsequently developed CRE-BSI. Platelet count (22.0×10³/μL) provided the strongest positive contribution, showing a 94.3% probability of BSI. Conversely, **Figure 8B** illustrates a prediction for a patient who did not develop BSI. A low imipenem or meropenem MIC (1 μg/mL) provided the strongest negative contribution, resulting in a 0.1% probability of BSI. These examples demonstrate how the model integrates multiple clinical parameters to provide individualized risk assessments.

## 4 Discussion

This study developed ML models for predicting subsequent BSI in CRE rectal carriers with excellent predictive performance. By combining data-driven ML with XAI, we identified multiple features contributing to CRE-BSI development and demonstrated an approach that addresses the limitations of traditional statistical methods.

Our findings revealed dynamic, time-dependent BSI risk profiles. For long-term (30-day) risk, the carbapenem MIC emerged as the strongest predictor, whereas for short-term (14-day) risk, the neutrophil count dominated. This temporal shift suggests that intrinsic microbial resistance primarily drives long-term risk, whereas acute host immune status is the critical determinant of imminent infection.

A key finding was the threshold effect of carbapenem MIC values. While CRE is defined by a MIC ≥4 μg/mL for imipenem or meropenem (Clinical and Laboratory Standards Institute, 2024), our results demonstrated that the risk of BSI was significantly elevated at concentrations ≥8 μg/mL and further increased at concentrations ≥16 μg/mL. This aligns with Liu et al. (2023), who reported increased BSI risk when both imipenem and meropenem MICs for rectal CRE exceeded 8 μg/mL. More notably, the 14-day model revealed an even higher threshold of ≥16 μg/mL, suggesting that short-term infection risk becomes apparent at higher resistance levels. This implies that rapid progression to BSI represents a higher acuity state, necessitating a greater burden of bacterial resistance to quickly overcome host defenses (Daikos and Markogiannakis, 2011). This temporal pattern is consistent with distinct features: short-term risk is driven by markers of immediate severity (high MIC, neutrophil count fluctuations), whereas long-term risk reflects cumulative exposure (Zhou et al., 2021). The 8 μg/mL threshold has important clinical and prognostic implications. Below this level, isolates remain susceptible to combination therapy with optimized dosing, which has been associated with greater mortality reduction compared with monotherapy (Daikos and Markogiannakis, 2011**;** Zhou et al., 2021). The emergence of this threshold in our BSI prediction model may reflect the prognostic impact of available antimicrobial options—patients with lower-MIC isolates have broader treatment choices and consequently better outcomes.

Our findings contrast markedly with the Giannella score (Giannella et al., 2014), which showed poor performance (AUROC <0.50) when applied to our data. This discrepancy likely arose from cohort differences—their study included only carbapenem-resistant *K. pneumoniae* (CRKP), while 26.3% of our cohort comprised non-CRKP. Additionally, our model identified respiratory CRE colonization as more important than number of colonization sites. Our results also differ from Liu et al. (2023), who reported linear increase in CRE-BSI risk with increasing age among hematologic malignancy patients. In contrast, we revealed a U-shaped relationship: below 73 years, risk increased with younger age and increased again in patients older than 85 years.

Unlike previous linear models limited to identifying independent risk factors, our ML approach captured complex non-linear patterns and feature interactions. Even the most important individual features—carbapenem MIC and neutrophil count—achieved only AUROC values of approximately 0.56–0.58 alone, confirming the necessity of considering multiple features.

Our study revealed complex interactions among features. BMI was positively associated with BSI risk, consistent with Rogne et al. (2020), who reported a 78% increase in BSI incidence at 30 kg/m² compared with 25 kg/m². In interaction with age, middle-aged patients with elevated BMI presented greater BSI risk than elderly patients. This may reflect the tendency of elderly individuals to have lower BMI due to age-related muscle loss and chronic diseases. Conversely, the calcium-age interaction showed the opposite pattern, with elderly patients demonstrating higher BSI risk. Our findings showed BSI risk increasing sharply below 8.0 mg/dL calcium, consistent with Chen et al. (2022). Age-related decline in calcium absorption likely explains the strong interaction between hypocalcemia and advanced age. These contrasting patterns show that a single feature can have opposing effects on BSI risk depending on interactions with other features—complexity that tree-based ML models with XAI can effectively capture.

The strengths of this study include a large multicenter dataset spanning 10 years with comprehensive routine laboratory tests. The combination of ML with XAI enables identification of complex non-linear relationships and feature interactions that traditional approaches cannot capture. Additionally, robust internal validation using 1, 000 bootstrap iterations ensured result stability and mitigated overfitting, providing reliable performance estimates.

However, our study has several limitations. First, we excluded patients who died or were discharged within the 30-day observation period. While this was methodologically necessary to ensure definitive outcome labels for supervised learning, it introduces selection bias toward patients with longer hospitalizations. Second, although we utilized data from multiple centers, geographic external validation using a completely independent dataset was not performed. Future prospective studies from different institutions are needed to confirm generalizability. Third, the retrospective design relying on CDW data may lack granular details on patient conditions or unrecorded interventions. Fourth, CRE-BSI was modeled as a binary outcome within two fixed follow-up periods rather than as a continuous time-to-event outcome; modeling the exact time to CRE-BSI is a direction for future work.Despite these limitations, risk stratification enabled by our model can guide clinical decision-making. The high concordance (84.7%) between rectal and blood isolate susceptibility supports using rectal CRE antimicrobial susceptibility results to guide empirical therapy in high-risk patients before blood culture results become available. Furthermore, the model enables dynamic risk reassessment by incorporating daily laboratory changes. This approach can inform antimicrobial stewardship decisions: high predicted risk may justify preemptive therapy, whereas low risk can prevent unnecessary antibiotic exposure.

## 5 Conclusion

This study demonstrates distinct time-dependent risk profiles in rectal CRE carriers. However, no single feature adequately predicts BSI, as patient features exist in complex interrelationships. By integrating XGBoost with SHAP-based explainability, this study demonstrated that multi-feature interactions can be effectively captured and interpreted, achieving predictive accuracy beyond traditional linear approaches. By providing individualized, interpretable BSI risk stratification, this approach can help clinicians decide when to initiate preemptive therapy in high-risk carriers and when to avoid unnecessary antibiotics in those at low risk, thereby supporting antimicrobial stewardship.

## Data Availability

The datasets presented in this study can be found in online repositories. The names of the repository/repositories and accession number(s) can be found below: Harvard Dataverse repository at https://doi.org/10.7910/DVN/DQ2CBX.
