# Supplementary material for: Machine learning-based prediction of bloodstream infection in rectal carbapenem-resistant Enterobacterales carriers: a multicenter retrospective cohort study
Source: Front Cell Infect Microbiol. 2026 Aug 6;16:1791859. doi: 10.3389/fcimb.2026.1791859 (PMC13490186; doi:10.3389/fcimb.2026.1791859)
Supplement: Supplementary file 1 [file DataSheet1.docx]

Supplementary Material

# Supplementary Table 1

173 Features collected from Clinical Data Warehouse (CDW).

| **Demographics & Vital** | | | | |
| --- | --- | --- | --- | --- |
| Age | Sex | | ABO group | |
| BMI | Highest Body Temperature | | Admission route | |
| Highest systolic blood pressure (SBP) | Lowest SBP/DBP | | Admission in COVID-19 | |
| Highest diastolic blood pressure (DBP) | Admission days before CRE (+) | | ICU days_before CRE (+) | |
| SBP/DBP at highest temperature | Pulse Rate at highest temperature | | ICU in f/u days | |
| Respiration Rate at highest temperature | Mental status | |  | |
| **Past history & Comorbidities** | | | | |
| Hypertension | Diabetes mellitus | | Tuberculosis | |
| CCI score | Asthma and COPD | | Past Cardiac disease | |
| qSOFA score | Chronic kidney disease | | Past Dyslipidemia | |
| COVID-19 | Hemiplegia | | Ileus | |
| Aspiration pneumonia | Dementia | | Sore | |
| Malignancy | Myocardial Infarction | | Smoking | |
| Acute Stroke |  | |  | |
| **CBC, Coagulation** | | | | |
| WBC count, Highest & Lowest | Platelet count, Highest & Lowest | | Neutrophil count, Highest & Lowest | |
| RBC counts | Hemoglobin | | RDW | |
| Prothrombin Time (PT) | PT, INR | | aPTT | |
| MPV |  | |  | |
| **Transfusion** | | | | |
| Any transfusion | | Packed RBC | | Fresh Frozen Plasma |
| Platelet concentrate | | Cryoprecipitate | |  |
| **Microbiology** | | | | |
| CPE | R: Imipenem or Meropenem | | one site colonized besides stool | |
| Carbapenemase_class (A, B, D) | R: Imipenem or Meropenem + Ertapenem | | two sites colonized besides stool | |
| Carbapenemase_KPC | MIC: Ertapenem | | other blood pathogen_MRSA | |
| Carbapenemase_NDM | MIC: Imipenem or Meropenem | | other blood pathogen_CRPA | |
| Carbapenemase_OXA 48 | MIC: IPM or MEM>8+ETP>4 | | other blood pathogen_bacteria | |
| Rectal CRE species | colonization_BAL | | other blood pathogen_candida | |
| other CRGNB in any sites | colonization_sputum | | other blood pathogen_GPC | |
| No. additional colonization sites | colonization_wound | | other blood pathogen_VRE | |
| No. other CRGNB in any sites | colonization_Urine | | other blood pathogen_CRAB | |
|  |  | | other blood pathogen_CRO | |
| **Chemistry** | | | | |
| Total calcium, Highest & Lowest | ALP | | Na, Highest & Lowest | |
| AST | ALT | | Cl, Highest & Lowest | |
| Total Cholesterol | LD | | K, Highest & Lowest | |
| GGT | BUN | | Creatinine | |
| Direct bilirubin | Total bilirubin | | Albumin | |
| **Drugs** | | | | |
| prior_any antibiotic | exposed_ chemo & radiation Tx | | exposed_ fluoroquinolone | |
| prior_ carbapenems | exposed_ Any antibiotic | | exposed_ aminoglycosides | |
| prior_ colistin | exposed_ carbapenems | | exposed_ oxyno cepha | |
| prior_ Tigecycline | exposed_ colistin | | exposed_ b lactam/binhibitor | |
| prior_ glycopeptides | exposed_ Tigecycline | | exposed_ corticosteroid | |
| prior_ Linezolid | prior_ oxyno cepha | | exposed_ glycopeptides | |
| prior_ fluoroquinolone | prior_ b lactam/b inhibitor | | exposed_ Linezolid | |
| prior_ aminoglycosides |  | |  | |
| **Procedures** | | | | |
| Dialysis | Rectal tube | | Dialysis | |
| Invasive Catheters | Tracheostomy | | Ventilator | |
| L-tube | PTBD | | TPN | |
| Gastrostomy, enterostomy | PEG | | Chest tube | |
| A line | PCD | | Cystostomy | |
| Foley catheter | Chemo port | | Colostomy | |
| EGD | C line | | Paracentesis | |
| Sigmoid colonoscopy | PICC | | Abdominal invasive procedure | |
| Endoscopic procedures | CPR | | Invasive hepatobiliary procedures | |

**Abbreviations:** **aPTT**, activated partial thromboplastin time; **ALP**, alkaline phosphatase; **ALT**, alanine aminotransferase; **AST**, aspartate aminotransferase; **BAL**, bronchoalveolar lavage; **BMI**, body mass index; **BUN**, blood urea nitrogen; **CCI**, Charlson comorbidity index; **COPD**, chronic obstructive pulmonary disease; **CPE**, carbapenemase-producing *Enterobactarles*; **CPR**, cardiopulmonary resuscitation; **CRAB**, carbapenem-resistant *Acinetobacter baumannii*; **CRE**, carbapenem-resistant *Enterobactarles*; **CRGNB**, carbapenem-resistant Gram-negative bacteria; **CRO**, carbapenem-resistant organism; **CRPA**, carbapenem-resistant *Pseudomonas aeruginosa*; **DBP**, diastolic blood pressure; **EGD**, esophagogastroduodenoscopy; **ETP**, ertapenem; **f/u**, follow-up; **GGT**, gamma-glutamyl transferase; **GPC**, Gram-positive cocci; **ICU**, intensive care unit; **INR**, international normalized ratio; **IPM**, imipenem; **KPC**, *Klebsiella pneumoniae* carbapenemase; **LD**, lactate dehydrogenase; **MEM**, meropenem; **MIC**, minimum inhibitory concentration; **MPV**, mean platelet volume; **MRSA**, methicillin-resistant *Staphylococcus aureus*; **NDM**, New Delhi metallo-beta-lactamase; **OXA**, oxacillinase; **PCD**, percutaneous catheter drainage; **PEG**, percutaneous endoscopic gastrostomy; **PICC**, peripherally inserted central catheter; **PT**, prothrombin time; **PTBD**, percutaneous transhepatic biliary drainage; **qSOFA**, quick sequential organ failure assessment; **RBC**, red blood cell; **RDW**, red cell distribution width; **SBP**, systolic blood pressure; **TPN**, total parenteral nutrition; **VRE**, vancomycin-resistant *Enterococcus*; **WBC**, white blood cell.

# Supplementary Table 2

Features with no impact on the CRE bloodstream infection prediction models

| **Category** | **Features** |
| --- | --- |
| **Demographics** | Admission during COVID-19 pandemic; Mental status: stupor |
| **Comorbidities** | Acute stroke; Asthma and COPD; Dementia; Hemiplegia; Myocardial infarction; Past cardiac disease; Past dyslipidemia; Smoking history |
| **Microbiology** | Carbapenemase: OXA-48; Other blood pathogen: MRSA; Other blood pathogen: bacteria; Other blood pathogen: *Candida* spp.; Other blood pathogen: CRAB; Other blood pathogen: CRPA; Other blood pathogen: GPC; Other blood pathogen: VRE; Rectal colonization: *Enterobacter* spp.; Rectal colonization: *Serratia marcescens*; Rectal colonization: Others; Number of other CRGNB in any sites; Positive colonization in urine; Positive colonization in wound |
| **Drugs** | Exposure to chemotherapy and radiation therapy; Exposure to aminoglycosides; Exposure to any antibiotics; Exposure to tigecycline; Prior use of aminoglycosides; Prior use of colistin; Prior use of oxyimino-cephalosporins |
| **Procedures** | Chest tube; Colostomy; Cystostomy; Foley catheter; Gastrostomy or enterostomy; Rectal tube; Sigmoid colonoscopy; EGD; Paracentesis; PCD; PEG; PICC; CPR; TPN |

**Abbreviations:** **COPD**, chronic obstructive pulmonary disease; **CPR**, cardiopulmonary resuscitation; **CRAB**, carbapenem-resistant *Acinetobacter baumannii*; **CRGNB**, carbapenem-resistant Gram-negative bacteria; **CRPA**, carbapenem-resistant *Pseudomonas aeruginosa*; **EGD**, esophagogastroduodenoscopy; **GPC**, Gram-positive cocci; **MRSA**, methicillin-resistant *Staphylococcus aureus*; **OXA**, oxacillinase; **PCD**, percutaneous catheter drainage; **PEG**, percutaneous endoscopic gastrostomy; **PICC**, peripherally inserted central catheter; **TPN**, total parenteral nutrition; **VRE**, vancomycin-resistant *Enterococcus*.

**Supplementary Figure 1.** Hierarchical clustering dendrogram of the 173 features. The x-axis indicates the cluster distance between features, and the y-axis lists the individual features. Distinct colors highlight separate clusters of highly correlated features.
